# Knowledge, and utilization of HIV self-testing, and its associated factors among women in sub–Saharan Africa: evidence from 21 countries demographic and health survey

**DOI:** 10.1186/s12889-024-19529-z

**Published:** 2024-07-23

**Authors:** Bewuketu Terefe, Mahlet Moges Jembere, Gashachew Bayleyegn Reda, Dejen Kahsay Asgedom, Solomon Keflie Assefa, Ayenew Molla Lakew

**Affiliations:** 1 Department of Community Health Nursing, School of Nursing, College of Medicine and Health Sciences, University of Gondar, Gondar, Ethiopia; 2 Department of Emergency, and Critical Care Nursing, School of Nursing, College of Medicine and Health Sciences, University of Gondar, Gondar, Ethiopia; 3 Department of Public Health, College of Medicine and Health Sciences, Samara University, Afar, Ethiopia; 4 Pawe Health Science College, Northwest, Ethiopia; 5 Department of Epidemiology, and Biostatistics, Institute of Public Health, College of Medicine and Health Sciences, University of Gondar, Gondar, Ethiopia

**Keywords:** Knowledge, And utilization, HIV self-testing, Women, Sub–Saharan Africa, Multilevel model

## Abstract

**Background:**

HIV Self-Testing (HIVST) holds great significance in the fight against the HIV epidemic in Sub-Saharan Africa (SSA). It offers a convenient and confidential option for individuals to know their HIV status and seek appropriate care and support. For women in this region, where stigma, discrimination, and lack of access to healthcare services are prevalent, HIVST can empower them to take control of their health and make informed decisions. However, no study in the region has been conducted on this topic. Hence, this study aimed to fill the evidence, and population gaps by identifying women’s HIVST knowledge, and utilization, and its associated factors in SSA.

**Methods:**

The data used were gathered from the most recent demographic and health surveys conducted in SSA nations between 2015 and 2022. We incorporated DHS data from 21 countries into our investigation. For our analysis, we used a weighted sample of 270,241 women overall was utilized. To handle both individual and community level factors, a multilevel logistic regression was used for the analysis. The adjusted odds ratio and its 95% confidence interval were then presented, and variables with univariate multilevel regression *p*-values of ≤ 0.25 and in multivariable multilevel logistic regression < 0.05 *p* value were considered significant factors of HIVST.

**Results:**

The overall prevalence of knowledge, and utilization of HIVST among women was about 2.17 (95% CI: 2.12, 2.23) only. Women aged 25–34 years old (AOR = 1.78, 95% CI: 1.65,1.92), and 35–49 years old (AOR = 1.33, 95% CI: 1.22,1.46), primary education(AOR = 1.25, 95%CI: 1.12, 1.38), and secondary/higher education (AOR = 3.08, 95% CI: 2.79, 3.41), poorer (AOR = 1.22, 95% CI: 1.08, 1.38), middle (AOR = 1.19, 95% CI: 1.06, 1.37), richer (AOR = 1.45, 95% CI 1.45, 1.64), and richest (AOR = 1.81, 95% CI: 1.59, 2.05), employed (AOR = 1.73 05% CI: 1.62, 1.85), mass media exposure (AOR = 1.39, 95% CI: 1.31, 1.49), knew modern contraception (AOR = 2.75, 95% CI: 1.84, 4.13), health facility delivery (AOR = 1.17, 95% CI: 1.02, 1.37), being from urban (AOR = 1.53, 95% CI: 1.63, 1.73), divorced or widowed (AOR = 77, 95% CI:1.13, 1.34), have more than one sexual partners (AOR =, 95% CI: 1.24, 1.41), heard about STIs (AOR 7.47 =, 95% CI: 5.16, 10.81), high community ANC coverage (AOR = 1.46, 95% CI: 1.31, 1.63), high community mass media (AOR = 1.37 95% CI: 1.21, 1.56), Central/Southern Africa (AOR = 0.66 95% CI: 0.59,0.74), and East Africa regions (AOR = 0.87 95% CI: 0.81,0.94) were associated with the knowledge and utilization of HIVST.

**Conclusions:**

The level of knowledge and utilization of HIVST among women in SSA was very low. To improve this situation, maternal health services can be enhanced. This can be achieved by facilitating institutional delivery, promoting access to modern contraception, increasing ANC coverage, empowering women’s associations, creating culturally respectful mass media content, and involving rural and economically disadvantaged women. By implementing these measures, we can enhance women’s knowledge and improve their use of HIVST.

## Introduction

Human Immunodeficiency Virus (HIV) is a serious public health issue around the world, with Sub-Saharan Africa (SSA) being the most afflicted [1, 2]. The Joint United Nations Program on HIV/AIDS (UNAIDS) 2023 report shows that about 39, and 1.3 million people were living with HIV, and were newly infected with HIV in 2022 [3]. Similarly, in the given year approximately 630, 000 people died of AIDS related illnesses [3]. Of them, two thirds came from Africa, where the majority of deaths are taking place [4]. The 2021 World Health Organization (WHO and UNAIDS) updated report estimated that about 36 million adult people living with HIV, 1.3 million new people acquiring HIV infection and 580,000 people dying from HIV related causes in 2020 [5]. The report also indicated that the African region remains the most severely attacked with the proportion of one in every 25 adults, which accounts two third of the globe [5, 6]. Hence, HIV/AIDS is the most fatal infectious disease, it accounted for 1.5% of deaths in 2019, globally however, the rate much higher in Africa approximately 28% across countries [7]. The alarming rate of HIV is an important public health issue for women’s and children’s of developing countries. For instance, the 2015 report showed that from a total estimation of 25.6 million people living with HIV, about 59% of them were women of reproductive age [8], and more than 1.2 million infants were exposed for HIV due to the comprehensive knowledge gap in the transmission of HIV according to UNICEF [9].

Any type of HIV testing in which a person gathers their own sample, conducts a quick, easy lab test, and learns the findings firsthand is known as HIV self-testing (HST). Self-testing has the potential to be a high impact, low cost, private, and user-empowering method that would complement scaling up testing [10]. Access to HST is crucial for prevention, treatment, and achieving the 95-95-95 target. However, by the end of 2016, nearly 30% of HIV-positive individuals were unaware of their status [11, 12]. HST and HIV testing through voluntary assisted partner notification are the two new HTS options that WHO recommends [11]. HIVST can increase the number of people who have tested, test more frequently, and encourage early HIV detection and, therefore, early treatment [13]. The following factors were identified by several literatures, as a contributor of increasing the transmission rate of HIV, and low knowledge about HIV such as, lack of awareness on HIV status, poor knowledge, education level, wealth index, failure to access to ART prophylaxis, poor adherence to ART, lack of clinic-based HIV education and counseling, types of residencies, being exposed to mass media, being exposed to HIV/AIDS education, having taken HIV test [14–22].

No prior study has assessed HIVST knowledge and use in the region. HIVST in SSA overcomes stigma, discrimination, and non-confidential testing, enhancing uptake over traditional methods [23]. The Self-Test Africa (STAR) initiative aims to distribute 4.7 million test kits in Africa. HIVST is more accepted by men than women, showing high uptake and potential for reaching undiagnosed individuals [24, 25]. However, challenges exist, including low knowledge of HIVST, the need for confirmatory tests, and the risk associated with testing strategies [25, 26]. Despite these challenges, HIVST is seen as a promising approach to improve access to testing and reach undiagnosed individuals in SSA [27].

Nearly two-thirds of all new HIV infections worldwide occur in Africa, with approximately 1.1 million infections reported in 2018. SSA countries such as Eswatini, Lesotho, Botswana, South Africa, Zimbabwe, Gambia, and others are among those with the highest HIV prevalence globally, ranging from 19.58 to 1.9% in the general population, according to WHO and various literature sources [28–31]. In some countries, among the total people living with HIV only 37% and 78.9% knowing their status and receiving ART respectively [32]. Based on evidence from the Gambian DHS 2019-20, only 27% of women and 28% of men have comprehensive knowledge about HIV [33]. About mother to child transmission of HIV also only 60% and 45% of women and men have comprehensive knowledge respectively [21, 33]. Despite efforts by international organizations, studies indicate low knowledge and attitudes towards HIV/AIDS among women in SSA, hindering progress in reducing the epidemic [21, 22, 34]. This may stem from inadequate understanding and skills related to HIVST and preventive strategies. Recognizing the importance of self-testing can help enhance its adoption, aligning with global targets such as the 95-95-95 initiative. This study aimed to assess the knowledge and utilization of HIVST among reproductive-aged women in SSA using recent survey data. By examining individual- and community-level factors, this study seeks to provide preliminary insights that can inform stakeholders and future research efforts.

## Methods

### Study setting, and period

The study utilized data from the most recent Demographic and Health Survey (DHS) conducted between 2015 and 2022 in 21 countries in SSA. In order to obtain a large and accurate sample that reflects the entire population and all its factors, the DHS conducts cross-sectional studies every five years on a global scale. These surveys have a significant sample size, are based on the population, and are representative of each nation on a national level.

### Data source, and study population

Specifically, in this study, researchers used individual recode (IR) files from these surveys. The DHS is a nationally representative survey conducted in over 90 low-income and middle-income countries. It collects data on important indicators such as breastfeeding, fertility, family planning, immunization, HIV/AIDS, child health, and nutrition [35]. For this study, a total of 270,241 reproductive-age women who had complete information on all the variables of interest from 21 SSA countries were included. The highest and lowest numbers of women were included in Malawi (24,008) and Zimbabwe (3,053), respectively (Table 1). The researchers followed the ‘Strengthening the Reporting of Observational Studies in Epidemiology’ (STROBE) statement while writing the manuscript [36]. The dataset used in this study is available for free download at: https://dhsprogram.com/data/available-datasets.cfm.

**Table 1:** Countries, survey years, and sample size of demographic and health surveys included in the analysis from 21 SSA countries

Countries	Survey year	Knowledge and use of HIVST (weighted sample)		Total sample (weighted)	Total sample (unweighted)
		No	Yes		
Burkina Faso	2021	17,617	42	17,659	17,659
Benin	2017/18	6,955	99	7,053	7,072
Burundi	2016/17	16,424	44	16,468	16,483
Côte d’Ivoire	2021	13,063	158	13,221	12,934
Cameron	2018	13,960	305	14,264	14,251
Gabon	2019/21	7,487	63	7,550	7,759
Gambia	2019/20	11,575	0	11,575	11,563
Guinea	2018	8,724	87	8,811	8,693
Kenya	2022	15,053	1,585	16,638	16,781
Liberia	2019/20	7,500	122	7,622	7,678
Madagascar	2021	14,962	55	15,017	14,942
Mali	2018	8,872	107	8,979	8,406
Mauritian	2019/21	13,728	178	13,906	13,782
Malawi	2015/16	23,783	225	24,008	24,072
Rwanda	2019/20	14,401	192	14,593	14,592
Sierra Leone	2019	13,999	603	14,603	14,434
Tanzania	2022	14,764	490	15,254	15,254
Uganda	2016	17,584	850	18,435	18,433
South Africa	2016	7,885	252	8,137	8,182
Zambia	2018	13,002	391	13,394	13,370
Zimbabwe	2015	3,026	27	3,053	2,986
Total		264,366	5,876	270,241	269,326

### Sample size determination and methods

All DHS surveys are conducted in developing countries, where statistics are often incomplete or outdated. Due to the typically outdated sampling frames, which are statistical categories based on national census data, DHS surveys employ a simple sampling design to ensure precise implementation and control of fieldwork. Therefore, most DHS surveys use stratified two-stage cluster sampling. This involves selecting primary sampling units (PSUs) in the first stage with a probability proportional to their size. In the second stage, a fixed number of households (or residential dwellings) is selected from a list of households obtained from the selected PSUs during an updating operation.

A PSU refers to a specific area or part of an area known as an Enumeration Area (EA). These EAs are based on the most recent population census and usually consist of multiple households. A comprehensive list of EAs provides information on their geographical location, rural-urban characteristics, total population, and total number of households. In addition, cartographic materials that clearly define the boundaries of these EAs are available. However, since a population census is conducted only once every ten years, information related to an EA, such as the number of households, is often outdated and requires updating. This updating process involves listing all households residing in the selected EAs and recording key information for each household, including the name of the household head, street address [37]. Following the listing of households, equal probability systematic sampling is used to select a specific number of households from inside the defined cluster [38].

### Definition of variables

#### Outcome variable

This study focused on HIVST as a dependent variable. To assess the outcomes, the researchers examined participants’ cumulative knowledge and usage of HIVST kits. The respondents were asked whether they were familiar with HIVST and whether they used these kits. The responses varied, with some stating that they had never heard of HIVST test kits, others indicating that they had tested using the kits, and some expressing knowledge of the kits but uncertainty about whether they had used them. For this study, women who were aware of and used HIV self-testing kits were considered to know and use HIVST. Therefore, those women who have the knowledge and have used HIVST were recorded as “Yes = 1”, if not, No = 0”.

#### Explanatory variable

This study considered approximately 20 explanatory variables, broadly grouped into individual-level (14 out of 20) and contextual (community-level) variables. Explanatory variables such as maternal age (15–24, 25–34, and 35–49 years old), marital status (single, married, other), number of sex partners (one and more than one), number of health visits in the past 12 months (once and more than once), age at first birth (15–19 and more than 19), women’s education (not educated, primary, and secondary/higher), wealth (poorest, poorer, middle, richer, and richest), awareness of STIs (no, yes), knowledge of modern contraceptive methods (no, yes), women’s employment status (no, yes), mass media exposure (no, yes), ANC follow-up (no, yes), place of delivery (health facility or home), gave birth (no, yes), residence (urban, rural), region (west, middle, and south, east), community-level ANC coverage (low, high), community illiteracy (low, high), community poverty (low, high), and community mass media exposure (low/high) were included as independent factors for knowledge and utilization of HIVST. These variables were not determined beforehand; instead, they were chosen based on the simplicity, theoretical relevance, and practical significance of HIV testing [39–42].

### Operational definitions for community level variables

Because the components at the community level could not be directly observed or recorded during the survey, their values were estimated using aggregated data from the individual records. Each component was estimated based on its distinct variable value following the methodology used in other studies. In this study, a cluster or primary sample unit in the dataset shared by a group of families was referred to as a community-level factor. The variables were then derived by combining the components at the individual, group, and community levels. The community variables included women’s education, media exposure, wealth, and ANC coverage. To facilitate understanding of the results, continuous community-level variables were categorized as low or high based on the mean or median value of their distribution [43–45].

#### Community’ women’s education

The educational achievement of women in the community is demonstrated by the median distribution of educational attainment. If the proportion of women in the community with at least a secondary education falls below the median (50%), it is considered low. Conversely, if it rises above the median (51–100%), it is considered high.

#### Community ANC coverage

The ANC coverage variable is based on individual ANC usage levels. If the percentage of women in the community who attended at least one ANC visit is between 50% and 100%, it is classified as high. If the percentage is between 0 and 49%, it is classified as low.

#### Community women’s media exposure

The variable for media exposure was based on how individuals responded to radio, books, or television. If fewer than 50% of women in the community were exposed to media, it was considered low. If between 50% and 100% were exposed, it was considered high.

#### Community women’s wealth

The variable for community wealth was derived using the same process for each household’s wealth index. For communities in the two lowest quintiles of wealth, it was considered high if there were between 61% and 100% women, and low if there were between 0% and 60% of women in the household.

### Data collection tools and procedure

Every five years, the DHS office collects data representative of the nation as a whole, focusing on the unique demographic and health challenges faced by each country. This was done through the use of five different surveys, including questionnaires for households, women, and men, as well as biomarkers and health institutions, including children. In this particular study, an individual questionnaire was used to gather information on the regional knowledge and use of HIVST, as well as the factors that contribute to HIVST. It is important to note that this investigation was conducted in accordance with the relevant statistical rules set forth by the DHS. The DHS program employs standardized methods and trained data collector professionals to ensure that the data collected are comparable across different countries. This includes the use of uniform questionnaires, manuals, and field procedures [46].

### Data quality control

Before data collection, a pretest was conducted on the DHS. Following the pre-test, a debriefing session was held with the fieldworkers involved. Any necessary changes to the questionnaire were made based on this session. Additional details can be found in the DHS guidance for more information on the data-collection process. You can access these details in the Guide to DHS statistics [46].

### Statistical analyses, and model building

The data were analyzed using Stata version 17.0 and Microsoft Excel 2019. The analysis was conducted in three steps. First, the prevalence of HIVST knowledge and utilization among women in SSA was graphically computed. A two-level multilevel mixed-effect binary and multiple logistic regression analysis was used to assess the impact of the explanatory variables on the knowledge and utilization of HIVST among women in SSA, taking into account the hierarchical nature of the data. Regarding the model parameter estimation for this investigation, a generalized linear mixed model (GLMM) was utilized, in which both random- and fixed-effect analyses were included in the linear predictor. Secondly, a univariate analysis was performed to calculate the proportions of HIVST across the explanatory and control variables, along with their significance levels (using a *p*-value of 0.25 as a cutoff point). To assess the presence of high correlations between the explanatory and control variables, a test for multicollinearity was conducted using the variance inflation factor (VIF). The results indicated no evidence of high collinearity (mean variance inflation factor (VIF) = 1.31, maximum VIF = 1.56, and minimum VIF = 1.02).

All variables that showed statistical significance in the binary regression analysis were included in a multiple multilevel logistic regression analysis. The analysis consisted of four models, namely Model 0, Model I, Model II, and Model III. Model 0 was an empty model with no explanatory or control variables. Model I included only the key individual-level explanatory variables. Model II included community-level control variables. Model III included all the explanatory variables from the individual and community levels. According to the model adequacy principles, this paper presents the results for Model III for reporting. The multilevel logistic regression analysis incorporated both fixed effects and random effects [47]. The fixed effects were presented as Adjusted Odds Ratios (AORs), while the random effects were assessed using the Intra-Cluster Correlation (ICC) [48]. Model comparison was conducted using the log-likelihood ratio (LLR) and Akaike Information Criterion (AIC) test. The model with the highest log-likelihood and lowest AIC was determined to be the best-fit (Table 3). Sampling weights are adjustment factors used to adjust for differences in the probability of selection and interviews between cases in a sample. These differences may be due to survey design and other factors. In demographic and health survey (DHS) surveys, the sample is often selected with unequal probability in order to increase the number of cases available and reduce sample variability for certain areas or subgroups. In such cases, weights must be applied to tabulations to accurately represent the population. Hence, all frequency distributions were weighted (v005/1,000,000), and the survey command “svy” in Stata was utilized to account for the complex sampling structure of the data in the regression analyses. The study used a multivariable multilevel logistic model to determine the factors associated with knowledge and utilization of HIVST among women. The best-fit model’s adjusted odds ratio (AOR) with a 95% confidence interval (CI) was reported in the final model.

**Table 2:** Individual and community-level factors associated with knowledge, and utilization of HIVST among women in 21 sub saharan African countries

HIVST knowledge and use variables	Null model	Model I	Model II	Model III
		AOR (95% CI)	AOR (95% CI)	AOR (95% CI)
Maternal age				
15–24		1		1
25–34		1.74(1.61,1.88)		1.78 (1.65,1.92) *
> 34		1.29(1.18,1.40)		1.33 (1.22,1.46) *
Maternal education				
Not educated		1		1
Primary		1.18(1.07,1.31)		1.25 (1.12,1.38) *
Secondary/higher		2.89(2.62,3.19)		3.08 (2.79,3.41) *
Women employed				
No		1		1
Yes		1.74(1.63,1.86)		1.73 (1.62,1.85) *
Wealth				
Poorest		1		1
Poorer		1.25(1.09,1.41)		1.22 (1.08,1.38) *
Middle		1.25(1.11,1.41)		1.19 (1.05,1.35) *
Richer		1.61(1.43,1.81)		1.45 (1.29,1.64) *
Richest		2.21(1.88,2.37)		1.81 (1.59,2.05) *
Mass media exposure				
No		1		1
Yes		1.38(1.29,1.47)		1.39 (1.31,1.49) *
Know modern method				
No		1		1
Yes		2.72(1.82,4.08)		2.75 (1.84,4.13) *
Marital status				
Single		1		1
Married		1.08(1.01,1.18)		1.06 (0.98,1.15)
Divorced/widowed		1.23(1.13,1.34)		1.23 (1.13,1.34) *
Place of delivery				
Home		1		1
Health facility		2.08(1.76,2.44)		2.03 (1.72,2.39) *
Sexual partner number				
One		1		1
> one		1.32(1.24,1.41)		1.32 (1.24,1.41) *
Heard about STIs				
No		1		1
Yes		6.97(4.82,10.07)		7.47 (5.16,10.81) *
Residence				
Urban			1.65(1.55,1.76)	1.63(1.53,1.73) *
Rural			1	1
Community ANC				
Low			1	1
High			1.53(1.36,1.72)	1.46 (1.31,1.63) *
Community education				
Low			1	1
High			1.27(1.11,1.45)	1.08 (0.96,1.23)
Community mass media				
Low			1	1
High			1.51(1.32,1.73)	1.37 (1.21,1.56) *
Region				
West			1	1
Central/ South			0.99(0.89,1.09)	0.66 (0.59,0.74) *
East			1.17(1.09,1.26)	0.87 (0.81,0.94) *
**Random parameters and model comparison**				
Community-level variance	0.94	0.63	0.64	0.55
ICC (%)	22.13	16.21	16.48	14.32
MOR (%)	2.51	2.13	2.14	2.02
PCV	Reference	32.98	31.92	41.49
Lok likelihood ratio (LLR)	-26,498	-24,520	-26,042	-24,432
DIC (-2LLR)	52,996	49,040	52,084	48,864
AIC	53,000	49,075	52,100	48,913

Where *= statistically significant variable at *P*-value < 0.05

## Results

### Sociodemographic characteristics of the study participants

Approximately 106,842 (39.54%) study women were between 15 and 24 years of reproductive age. Regarding marital status, more than half of the women, 130,362 (48.24%) were married. Regarding place of residence, 160,289 (59.99%) were from rural areas. In terms of educational status, 107,925 (39.94%) women had secondary or higher education. Regarding the wealth index, 66,483 (24.6%) women were from the richest households. Regarding employment status, 174,633 (64.62%) were currently employed. Furthermore, regarding maternal health services, 262,100 (96.99%) women had at least one ANC visit, 248,272 (91.87%) gave birth at health institutions, 261,438 (97.51%) knew about modern contraceptive methods, and 255,793 (94.65%) had heard about STIs through different opportunities. Approximately 143,909 (53.25%) women had mass media exposure (listening to the radio, watching television, or reading magazines or newspapers). However, only 160,368 (59.34%) gave birth at the age of more than 19 years, and 62,218 (23.02%) had more than one health facility visit in the past 12 months. In contrast, 169,473 (62.71%) participants had more than one sex partner. Regarding birth history, 194,963 (72.14%) had given birth. Regarding community-level variables, 136,827 (50.59%) had high ANC coverage, 140,984 (78.86%) had high community women’s education, 138,646 (47.21%) had high community media exposure, and 176,663 (65.37%) had high wealth. The majority, 175,563 (64.97%) of the respondents were from the Eastern region (Table 2).

**Table 3:** Sociodemographic, and maternal health related characteristics of HIVST among women in 21 sub saharan African countries (*n* = 270,241)

HIVST knowledge and use	Weighted frequency	Weighted percentage
Maternal age		
15–24	106,842	39.54
25–34	83,436	30.87
> 34	79,964	29.59
Maternal education		
Not educated	69,488	25.71
Primary	92,829	34.35
Secondary/higher	107,925	39.94
Women employed		
No	95,609	35.38
Yes	174,633	64.62
Wealth		
Poorest	44,852	16.60
Poorer	48,726	18.03
Middle	51,844	19.18
Richer	58,336	21.59
Richest	66,483	24.60
Mass media exposure		
No	126,333	46.75
Yes	143,909	53.25
Know modern contraceptive method		
No	6,671	2.49
Yes	261,438	97.51
Marital status		
Single	80,893	29.93
Married	130,362	48.24
Divorced/widowed	58,986	21.83
At least one ANC visit		
No	8,141	3.01
Yes	262,100	96.99
Place of delivery		
Home	21,970	8.13
Health facility	248,272	91.87
Age at first birth in years		
< 20	109,873	40.66
≥ 20	160,368	59.34
Number of health facility visit in the past 12 months		
Once	208,024	76.98
More than once	62,218	23.02
Sexual partner number		
One	100,768	37.29
More than one	169,473	62.71
Heard about STIs		
No	14,449	5.35
Yes	255,793	94.65
Ever gave birth		
No	75,278	27.86
Yes	194,963	72.14
Residence		
Urban	109,953	40.69
Rural	160,289	59.31
Community wealth		
Low	93,579	34.63
High	176,663	65.37
Community ANC coverage		
Low	133,414	49.37
High	136,827	50.63
Community education		
Low	129,257	47.83
High	140,984	52.17
Community mass media		
Low	131,595	48.70
High	138,646	51.30
Region		
West	64,727	23.95
Central/ South	29,952	11.08
East	175,563	64.97

### Knowledge, and utilization of HIVST among women

**Fig. 1:**
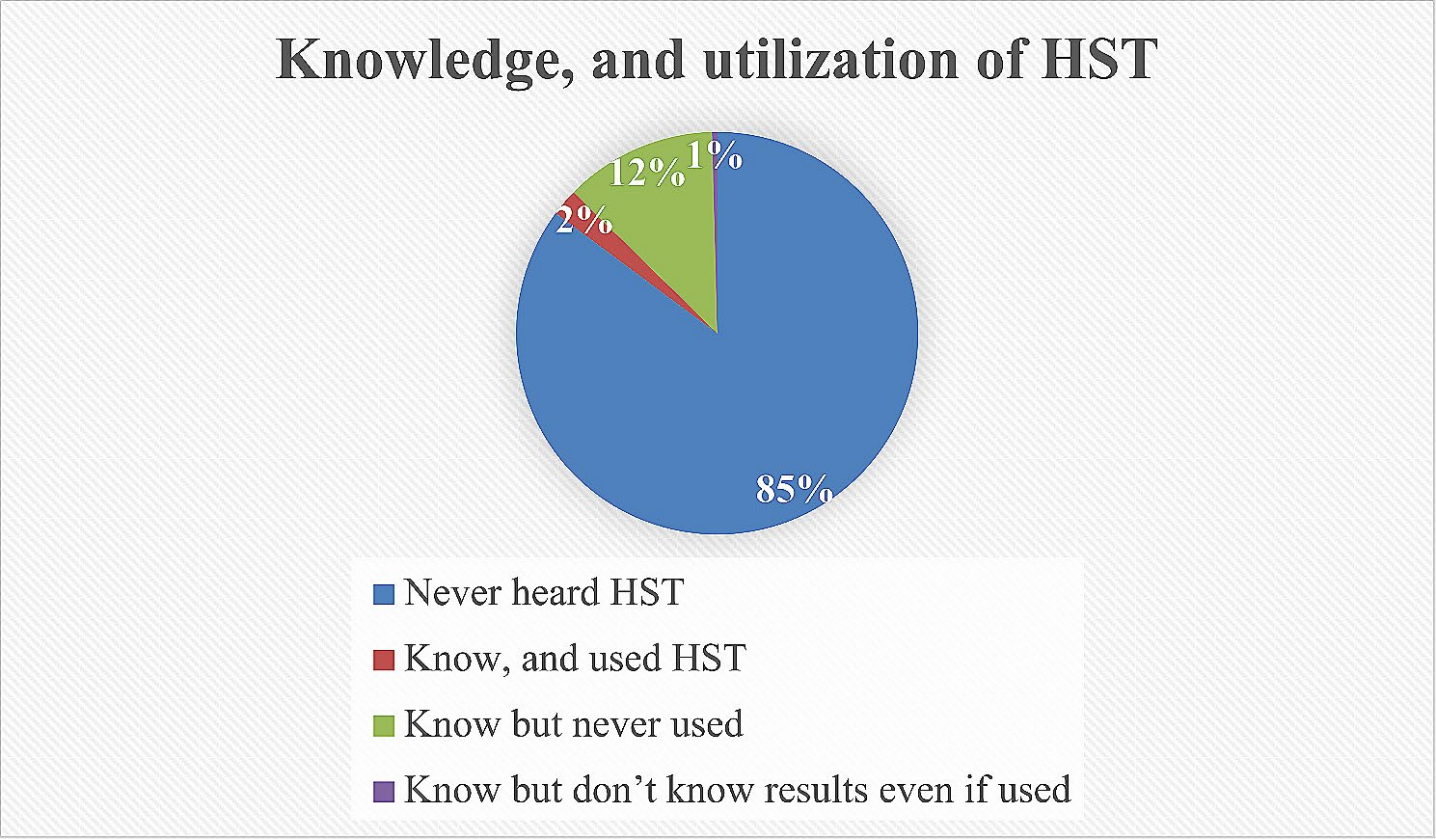
Shows knowledge, and utilization of HIV self-testing among women in sub–Saharan African countries

The overall proportion of knowledge and appropriate utilization of HIVST among women was approximately 2.17% (95% CI: 2.12, 2.23). On the other hand, about 85.16% (95% CI, 85.03, 85.30) of them did not even hear anything about HIVST. Similarly, although about 12.25% (95% CI, 12.13,12.37) women knew about HIVST, they had never utilized the kit by themselves because of a lack of appropriate knowledge about it. Finally, the remaining small proportion women knew test kits, however, they did not realize the results of the test. In general speaking about 97.83% (95% CI, 97.77,97.88) of women did not show both knowledge and utilization of HIVST. The three highest rates of HIVST knowledge and utilization among women were in Malawi (8.89%), Burundi (6.09%), and Burkina Faso (6.34%). The lowest proportions of HIVST were observed in Zimbabwe (1.13%), Benin (2.61%), and Ghana (2.79%) (Fig. 1).

### Random effects analysis of HIVST among women

The random-effects model revealed a significant clustering of knowledge and use of HIVST among women across communities (community-level variance = 0.94). The amount of variability in achieving knowledge and use of HIVST among women explained by cluster variation was 22.13% (95% CI: 20.09, 24.17) based on the estimated intraclass correlation coefficient (ICC). Individual-level variations explained 77.87% of the variability in women’s knowledge and utilization of HIVST. When a woman moves from an area with a low probability of knowledge and utilization of HIVST to an area with a high probability of knowledge and utilization of HIVST, there is a 51% increased probability of having better knowledge and utilization of HIVST, as indicated by the median odds ratio (MOR = 2.51; 95% CI: 2.18, 2.87). The amount of variability explained by individual-level factors, community-level factors, and both individual- and community-level factors was 32.98%, 31.92%, and 41.49%, respectively. Based on the Likelihood Ratio (LLR) test (LLR test mixed-effect versus the classical logistic model, *P* = 0.00001), the data suggest that models that consider the clustering effect are a better fit. Among these, Model IV, which included individual- and community-level factors, was the best. This model had the smallest deviance and largest LLR, and the Akaike Information Criterion (AIC) value indicated that it was a better fit than the other models. The associated factors were reported based on this model. Therefore, a mixed-effects model was considered to be an appropriate analytical model for the proposed topic (Table 3).

### Fixed effect analysis of associated factors with HIVST among women

Women aged 25–34 years (AOR = 1.78, 95% CI: 1.65–1.92) and 35–49 years (AOR = 1.33, 95% CI: 1.22–1.46) demonstrated better knowledge and utilization of HIVST compared to women aged 15–24 years. Women with primary education (AOR = 1.25, 95% CI: 1.12–1.38) and secondary/higher education (AOR = 3.08, 95% CI: 2.79–3.41) had higher odds of knowledge and utilization of HIVST compared to uneducated women. Regarding household wealth, women from poorer (AOR = 1.22, 95% CI: 1.08–1.38), middle (AOR = 1.19, 95% CI: 1.06–1.37), richer (AOR = 1.45, 95% CI: 1.29–1.64), and richest (AOR = 1.81, 95% CI: 1.59–2.05) households had higher odds of HIVST knowledge and utilization than women from the poorest households. Employed women had higher odds (AOR = 1.73, 95% CI: 1.62–1.85) of HIVST knowledge and utilization than unemployed women did. Exposure to media (television, radio, or newspapers) was associated with higher odds (AOR = 1.39, 95% CI: 1.31–1.49) of HIVST knowledge and utilization compared to those not exposed to media. Women who were aware of modern contraception (AOR = 2.75, 95% CI: 1.84–4.13) and those who gave birth at health institutions (AOR = 1.17, 95% CI: 1.02–1.37) had higher odds of HIVST knowledge and utilization. Women from urban areas had higher odds (AOR = 1.63, 95% CI: 1.53–1.73) of HIVST knowledge and utilization compared to rural women. Divorced or widowed women had higher odds (AOR = 1.23, 95% CI: 1.13–1.34) of HIVST knowledge and utilization than unmarried women. Women with more than one sexual partner had higher odds (AOR = 1.32, 95% CI: 1.24–1.41) compared to those with one partner. Women aware of STIs had significantly higher odds (AOR = 7.47, 95% CI: 5.16–10.81) of HIVST knowledge and utilization compared to those who were unaware of STIs. Community-level factors such as high ANC coverage (AOR = 1.46, 95% CI: 1.31–1.63) and high community mass media exposure (AOR = 1.37, 95% CI: 1.21–1.56) were associated with higher odds of HIVST knowledge and utilization. Women living in the Central/southern (AOR = 0.66, 95% CI: 0.59–0.74) and East Africa (AOR = 0.87, 95% CI: 0.81–0.94) regions had lower odds of HIVST knowledge and utilization than those in the western region (Table 3).

## Discussion

This nationwide, community-based cross-sectional study was conducted across 21 SSA countries to assess the knowledge and usage of HIVST, as well as its possible associated factors among reproductive-age women. After controlling for confounders and conducting the appropriate statistical analyses, both individual and community-level factors were found to be associated with the variable of interest in the final model. Explanatory factors such as older age, formal education, higher household wealth indexes, media exposure, knowledge about modern contraception, divorced/widowed marital status, having multiple sexual partners, institutional delivery, awareness of STIs, urban residence, high ANC coverage, and coming from countries in East and Central/Southern Africa were independently associated with the knowledge and utilization of HIVST among women in SSA.

Older women have been found to have better knowledge and usage of HIVST compared to young women. A study in Kenya revealed that while awareness of oral HIVST was low (19%) among young adults, willingness to use HIVST kit was high (75%), with women, older participants being more willing to use HIVST kits [49]. Similarly, research in Malawi and South Africa found that midlife-older adults expressed a marked preference for access to HIVST, and they were more open to self-testing compared to younger adults [50, 51]. The reasons for this difference in knowledge and usage could be attributed to various factors such as education, life experience, and awareness of personal risk. Older women may be more exposed to HIV/AIDS information over their lifetime, leading to better knowledge and understanding of the importance of testing [34]. Furthermore, they may be more proactive in seeking testing due to greater awareness of the risks associated with HIV. This highlights the need for targeted messaging and services to promote HIVST among young girls, and addresses the unique challenges and unmet needs of different age groups in the context of HIV/AIDS.

Women with primary education had 1.25 times higher odds of having HIVST knowledge and use than those with no formal education. Similarly, women with secondary or higher education had 3.08 times higher odds of having HIVST knowledge and use than those with no formal education. This study is consistent with a study conducted in South Africa, China, [52–56]. The possible justification for this might be that education contributes to knowing one’s HIV status and using HIV services; however, those women who live in rural areas had an 11% reduction in HIVST knowledge and use when compared with those women who lived in urban areas. This result is congruent with a study conducted in South Africa, Malaysia, and Philippines [34, 52, 57, 58]. A possible reason for this could be that rural populations are not served with healthcare services and are not accessible to appropriate health information.

Mothers who have ANC follow-ups, deliver at health institutions, and are knowledgeable about modern contraceptives have higher odds of better knowledge and usage of HIVST than their counterparts. Several studies have shown that the ANC platform offers a unique opportunity to increase HIV testing by distributing self-testing kits, and knowledge [21, 59]. For example, a study in Kenya found that giving ANC mothers test kits and improved male invitation letters increased the likelihood of male partner testing by twelve times [60]. Another study in South Africa evaluated the programmatic implementation of partner-delivered self-testing through ANC attendees and found a high proportion of partners offered and using the delivered HIV self-test kits [61]. These findings suggest that ANC services can be effectively utilized to increase HIV testing and self-test kit distribution, especially among male partners, ultimately contributing to better knowledge and usage of HIVST among mothers receiving ANC services [61]. Additionally, the World Health Organization emphasizes the importance of integrating HIV prevention, including testing and linkage to antiretroviral therapy, within contraceptive and reproductive health services, especially due to the high incidence of HIV among women [62]. Furthermore, women with greater decision-making power have been found to have more knowledge and use of HIV test kits [63]. These findings suggest that the combination of ANC services, knowledge of modern contraceptives, and institutional delivery plays a significant role in increasing the knowledge and usage of HIV self-tests among mothers.

Women who had mass media exposure had 1.39 times higher odds of having good knowledge and use of HIVST than those without mass media exposure. This study is consistent with a study conducted in South Africa [64]. A possible justification might be that women who are more exposed to mass media get more information about HIV/AIDS and become more aware. This study is not consistent with a study conducted in Malaysia [57]. A possible reason for this could be the sample size, study design, and study period. Additionally, sociocultural factors and the content of media messages could play a role. Women might be more likely to absorb and apply information about HIVST in contexts where media messages are more comprehensive and culturally relevant. Conversely, in settings where media coverage is less focused on health issues or where competing health concerns dominate the public discourse, the impact of mass media exposure on HIVST knowledge and use may be diluted. Women who had more than one sexual partner had higher odds of having good knowledge and use of HIVST than those who had one sexual partner. This study is consistent with a study conducted in Uganda and China [54, 65]. This study is not in line with a study conducted in Malaysia [57].The possible reason may be sample size, study design, and study period.

Divorced or widowed mothers showed higher odds of having better knowledge and use of HIVST than single women. According to a study conducted in rural Zimbabwe, widowed/separated/divorced women were more likely to have heard of HIVST than single women [66]. However, this study did not provide an explanation for this finding. It might be that divorced or widowed women do not have to be pressured or dominated by anyone to be tested for HIV. In addition, because they might engage in various inappropriate sexual activities, the chances of being diagnosed with HIV increase. Another study conducted in Northern Thailand found that marriage was associated with higher knowledge of HIVST [67]. The reason for this association is not clear, but it may be related to the fact that married women are more likely to have children and may be more concerned about their health and that of their families [68]. Overall, more research is needed to understand why divorced or widowed mothers are more likely to have better knowledge and use of HIVST than single women.

Women who were poorer (AOR = 1.22), middle income (AOR = 1.19), richer (AOR = 1.45), and richest (AOR = 1.81) had higher odds of having good knowledge and use of HIVST than those from the poorest households. This study is in line with a study conducted in South Africa and China [52, 65, 69, 70]. A possible reason might be that those women with high income gained access to health information and better services, leading to greater health awareness due to the use of mass media and other information means.

Women who heard about STIs had higher odds of having good knowledge and use of HIVST than those who had not heard about STIs. This study is consistent with a study conducted in Uganda and China [54–56, 70]. Possible reasons for this association could include heightened health consciousness among individuals who are already aware of STIs, leading them to actively seek information about HIV and its testing options. Moreover, cultural attitudes towards sexual health and openness to discussing STIs may influence how readily women engage with information related to HIVST. Additionally, the quality and accessibility of healthcare services in regions with higher STI awareness could contribute to a more informed community, where individuals are more likely to adopt new health technologies, such as HIVST.

Regarding regional variation, women in West Africa had higher odds of having access to and exhibiting a higher level of acceptance of HIVST than women in East or Central Africa. Studies conducted in countries such as Benin, Côte d’Ivoire, Mali, and Senegal have demonstrated a remarkably high level of acceptability for HIVST among female sex workers and the general population in West Africa [71–73]. The introduction of HIVST in West Africa has been shown to help overcome some of the barriers to testing encountered in other parts of Africa [71, 74]. This increased access and acceptability in West Africa can be attributed to the implementation of programs and initiatives that promote HIVST in the region as well as the feasibility and effectiveness of self-testing initiatives. However, it is important to note that the specific reasons for the variations in access and acceptability between West Africa and other regions of the continent may differ, necessitating further in-depth research and analysis.

### Strength, and limitations of the study

The strength of this study lies in its use of nationally representative surveys from 21 SSA nations to evaluate the knowledge and utilization of HIVST among women and the factors that influence it at both the individual and community levels. Therefore, we believe that our findings can be applied to these countries as well as other SSA nations. Another significant strength is the incorporation of various possible factors into the outcome variables by using an advanced mixed-effects model approach. This model allowed us to uncover hidden and unexplored factors that may affect women’s ability to achieve the desired outcome. In addition, the study data were gathered using conventional and verified data-gathering procedures, which adds to the strengths of this study. Finally, being the first of its kind, this study will serve as a foundation for future researchers, policymakers, and health professionals. However, this study had some limitations. First, due to its cross-sectional nature, a cause-and-effect link cannot be established. Second, reliance on self-reported statistics in the DHS may introduce bias due to memory issues. Furthermore, certain important factors such as accessibility to treatment, sociocultural-related factors, issues related to health professionals, and support programs were not included in the analysis. Moreover, the use of surveys conducted at different locations within the chosen countries was necessitated by the data constraints and availability. It is also important to consider the limitations of the cross-sectional design. Finally, DHS surveys in different countries were conducted at varying time periods, ranging from 2015 to 2022. This temporal difference in data collection across countries makes it difficult to interpret the combined estimates of HIVST knowledge and utilization.

## Conclusions

Achievement of knowledge and appropriate utilization of HIVST among women in SSA has been found to be very low. In addition to the knowledge deficit, the lack of appropriate utilization of HIVST was another challenge for women to conduct HIVST after they heard of it. In the final model analysis, various factors independently contributed to the knowledge and use of highly effective short-term HIVST among women in SSA. These factors include being an older woman, having a formal education, having a higher household wealth index, being exposed to the media, knowing about modern contraception, being divorced or widowed, having multiple sexual partners, delivering in a healthcare institution, being aware of STIs, living in an urban area, having high community ANC coverage, and coming from countries in East and Central/Southern Africa regions.

Efforts should be made to overcome the barriers that hinder women’s knowledge and use of HIVST to slow the spread of HIV/AIDS. Therefore, all parties involved in HIV/AIDS prevention and control, regardless of nationality, should consider the aforementioned factors. There are several benefits of helping economically disadvantaged women, including enhancing HIVST education within the community, establishing community-based infectious disease associations, and raising awareness. Additionally, by improving maternal health services, such as providing institutional delivery, modern contraception, ANC coverage, and empowering women, as well as creating mass media content that respects the cultural traditions of the people, and providing free drugs in ample quantities, it is possible to increase women’s knowledge and improve their attitudes towards HIVST. As a result, women’s health will be preserved and the prevalence of HIV/AIDS will decrease. Moreover, by implementing HIV prevention programs that specifically target young women, access to healthcare coverage involving rural women can be expanded in community-wide initiatives. It is also crucial for upcoming researchers in this field to consider variables related to culture and employ spatial epidemiological approaches in their investigations.

## Data Availability

All data concerning this study are accommodated and presented in this document. The detailed data set can be freely accessed from the www. dhsprogram.com website.
